# Phenolic Compounds and Antioxidant Activity: Analytical Methods and Current Knowledge—A Review

**DOI:** 10.3390/mps9020060

**Published:** 2026-04-03

**Authors:** Miroslav Lisjak, Marija Špoljarević, Jelena Ravlić, Zdenko Lončarić, Lucija Galić

**Affiliations:** Faculty of Agrobiotechnical Sciences Osijek, Josip Juraj Strossmayer University of Osijek, Vladimira Preloga 1, 31000 Osijek, Croatia; mlisjak@fazos.hr (M.L.); zloncaric@fazos.hr (Z.L.)

**Keywords:** DPPH, FRAP, ORAC, HPLC, oxidative stress, detection methods

## Abstract

Phenolic compounds are plant-derived antioxidants crucial for human health and food preservation. Their bioactive potential including anti-inflammatory, antimicrobial, and anti-carcinogenic properties makes them a vital focus in nutritional, pharmaceutical, and agricultural research. This review critically evaluates the methodologies for their extraction, detection, and quantification to accurately assess antioxidant activity. Oxidative stress in biological systems and food matrices necessitates accurate analytical methodologies for assessing antioxidant behavior, which include both in vitro, in vivo and ex vivo approaches. Sample pretreatment and extraction techniques are critical for reliable analysis and vary depending on the matrix, compound polarity, and target phenolic subclass. We compare conventional extraction techniques (Soxhlet, maceration) with advanced methods like ultrasound-assisted, microwave-assisted, and supercritical fluid extraction. Detection methods reviewed include spectrophotometric assays (e.g., DPPH, FRAP, ORAC), electrochemical sensors, and chromatographic techniques (e.g., HPLC, HPLC−MS). While each method has distinct advantages, a lack of standardization remains the primary challenge, driven by variations in protocols and the vast chemical diversity of phenolics. This review underscores the critical need for integrated, standardized approaches to ensure the accurate and comparable evaluation of antioxidant activity in research and industry.

## 1. Introduction

The importance of oxidation in the body and in food is widely recognized. Oxidative metabolism is crucial for the survival of cells [1,2]. The byproduct of this dependency is free radicals and other reactive oxygen species that result in oxidative changes and breakdown [3]. Increasing research points to the role of such species in different types of in vivo control mechanisms. An over-production of free radicals triggers the protective enzymatic response of superoxide dismutase, catalase and peroxidase and results in fatal and damaging cellular responses (e.g., apoptosis), the oxidation of membrane lipids, cellular proteins, DNA and enzymes, and the disabling of cellular respiration [4,5]. Reactive oxygen species affect cellular signaling pathways in various ways. Oxidation can also affect food, where it is one of the main causes of chemical spoilage, resulting in the decay and/or deterioration of nutritional quality, color, taste, texture, and the safety or wholesomeness of food [4,6]. It is estimated that half of the world’s fruit and vegetable crops are lost due to inadequate post-harvest storage, which causes chemical changes, i.e., spoilage. Antioxidants have protective defences against excessive oxidation and studying the importance of measuring oxidative responses is well documented. There are two main approaches used to assess antioxidant behaviour and these two approaches reflect the focus of the activity in foods or the bioactivity as relates to humans [3]. In the case of food systems, assessing the effectiveness of antioxidants is necessary to ensure the protection of food from oxidative spoilage. A subcategory includes the measurement of activity in food, particularly fruits, vegetables, and beverages, but with the aim of predicting nutritional effects in vivo [7,8]. Compared to plant foods, meat, milk, and cheese have phenolic compounds at much lower concentration, making phenolic analysis in these foodstuffs very complicated and analytically challenging. This is partly because of matrix complexity and interference from high-protein and high-fat constituents. Other than the compounds present in the animal body, the animal’s feed (such as grass), the additives in the process, and the spices or smoked additives in the process also contribute phenolic compounds [9], or formation during maturation, as in aged cheeses [10]. Oxidation is one of the most important processes, creating free radicals in food, chemicals, and even in living systems, often through Fenton reactions. Fenton reactions represent the reactions of hydrogen peroxide and a metal in the presence of a proton, where hydroxyl radicals and water are formed [11]. Hydroxyl radicals can further react with hydrogen peroxide to form hydroperoxyl radicals, which drive food spoilage, material degradation, and contribute to numerous human disorders [12]. Low-dose antioxidants powerfully shield materials from oxidative decay [13]. Long used to stabilize industrial materials, antioxidants are now a major focus in medical therapy [14].

In humans, oxidative stress occurs due to an imbalance between reactive oxygen species (ROS) and the body’s defense and repair systems. Some of the body’s endogenous defenses include superoxide dismutase, catalase, and glutathione peroxidase, along with vitamin E, uric acid, and serum albumins. Besides these cellular stress defense species, the intake of antioxidants from food is also vital [15,16]. Various methods for measuring antioxidant activity, especially in relation to lipid oxidation, should be distinguished from the related processes of measuring antioxidant concentration. However, it can be said that these are two related methods, given that antioxidants often show pro-oxidant effects at higher concentrations [17]. The term “activity” applied to antioxidants needs to be clarified as it can have a multitude of meanings. Efficiency hinges on reaction speed, environment (water vs. oil), potency, and teamwork with other antioxidants [3,18]. If specific materials cannot be tested directly, extracts are screened using methods like TLC to identify active antioxidants. Spray reagents can further reveal their chemical class (e.g., phenols) and functional mechanism [18]. Lab screening is only a starting point; true effectiveness must be proven in the final substrate. In living systems, factors like metabolic transformation and competing enzymes can drastically alter an antioxidant’s real-world impact [19]. Current plant research is often oversimplified, stripping away the natural structures that define how well an antioxidant actually works. Because these structures and various testing methods differ so much, comparing data across studies remains a significant challenge [20].

## 2. Review Methodology

A systematic narrative review was executed on current methodologies for assessing phenolic compounds and antioxidant activities on the basis of Web of Science, Scopus, and PubMed. Boolean searches of the keywords polyphenols, antioxidant capacity combined with different analytical methods, and DPPH and/or HPLC were used. We included only English peer-reviewed articles from January 2000 to January 2025 on the extraction, determination, or quantification of phenolic compounds from plant sources and some of the pre-2000 articles on methods. Because we were searching for a specific time period, we included the seminal “classic” publications that are recognized as the originators of certain methodological protocols (i.e., methods by Singleton, Brand-Williams, and Benzie).

Data on the mechanisms of the assays, the matrices used, their limitations and QC were extracted and analyzed from the full texts after title and abstract screening and were summarized in decision matrices.

## 3. Types of Extraction of Phenolic Compounds from Plants

Accurate determination of phenolic content hinges on the effectiveness of preliminary processing. Selection of a technique should be guided by the nature of the matrix: solid plant matter (seeds, bark), high-moisture foods (fruits, vegetables), or complex liquids (milk, wine) [17,18]. The target phenolics’ chemical structure dictates the most effective method. For instance, polar solvents usually recover simple phenolic acids well, whereas more complex phenolics, like tannins or esterified phenolics, need covalent bond-breaking hydrolysis (acid or alkaline) with cell wall polysaccharides. For high-fat matrices (like seeds or dairy), a pre-treatment defatting step with n-hexane is essential to minimize lipid interference during extraction. Because phenolic acids vary so much in chemistry and concentration, no universal pretreatment method exists [21]. Selecting an appropriate pretreatment based on molecular characteristics is crucial, as the analyst is responsible for overseeing how multi-stage processing affects the data [22]. Complexes with proteins, carbohydrates, or other elements prevent the complete extraction of some phenols. For some preparation techniques, plant samples need to be freeze-dried, air-dried, or oven-dried [23]. As an example, shade-dried samples can yield more phenolic compounds than those samples that have been oven-dried. Dry samples are milled or crushed to obtain a certain particle size. In contrast, liquid samples are subjected to a series of processes which include centrifugation, filtration, and purification within separation systems [24]. Comprehensive sample pretreatment combining particle size reduction with defatting is critical for overcoming matrix barriers and ensuring the highest possible concentration of extracted polyphenols [25,26,27].

After sample preparation, the subsequent and equally important process is the complete extraction of phenolic compounds. The extraction of phenolic acids most commonly employs solvents which can be organic [28]. Extraction parameters such as time, temperature, solvent-to-sample ratio, number of sample repetitive extractions, and solvent type can all affect yield. In addition, the sample type, and the active compounds and phenolic acid composition within different plant species can affect the optimal recovery of phenolic acids [16]. The choice of extraction solvent, such as water, acetone, ethyl acetate, alcohols (methanol, ethanol, and propanol), and their mixtures, will affect the number of phenols extracted. For example, a high content of phenolic acids can be extracted from leaves using water, while an 80% ethanol solution is required for extracting phenols from wheat bran [29]. For instance, a study regarding the extraction of phenolic compounds from the aerial parts of *Potentilla atrosanguinea* showed that 50% ethanol was more efficient than the other solvents, whether they were pure, 50% diluted, or included methanol or acetone. On the other hand, grapevine waste, i.e., grapes (*Vitis vinifera*) and sunflower meal (*Helianthus annuus*) showed the highest phenolic compound yields when extracted with pure methanol and 80% acetone. These variances could be the result of the structural and functional properties of the phenolic constituents of the studied plants [30,31].

Successful phenolic extraction requires optimizing physical parameters and employing hydrolysis to liberate matrix-bound compounds, while carefully balancing increased solubility against the risk of oxidative degradation [32]. It is important to conduct sample preparation and separation of interfering substances for accurate quantification of phenolic acids, but the extraction method is the most critical factor in determining the separation and recovery of phenolic acids. Extraction is influenced by the sample characteristics, particle size, nature of the solvent, and the extraction methods employed. Common methods of extracting phenolic acids from solid samples include Soxhlet extraction, heated reflux extraction, and maceration. Soxhlet and heated reflux extraction methods are typically performed at 90 °C for several hours, while maceration can take several days at room temperature [33,34]. These methods are simple, require relatively inexpensive apparatus, and result in appropriately high extraction rates but it depends on how many samples you have and the amount of plant material. In addition to these conventional techniques, alternative methods such as Ultrasound-Assisted Extraction (UAE) have also gained attention for their efficiency and reduced extraction time [35]. UAE is ideal for soft tissues (leaves, pulp) and sensitive, thermolabile compounds like anthocyanins. Ultrasound-Assisted Extraction (UAE) method uses ultrasonic waves (>20 kHz) to create cavitation bubbles that collapse and disrupt plant cell walls, releasing their contents. UAE is a simple, inexpensive, and rapid technique that improves mass transfer and solvent penetration. While effective, its efficiency can be lower than some thermal methods [21]. Microwave-Assisted Extraction (MAE) uses microwave energy (300 MHz to 300 GHz) to heat the solvent and water within the plant cells and is most efficient for recovering stable phenolic acids from hardened or dried matrices (roots, seeds) through rapid cell rupture. This creates internal pressure that ruptures the cells, releasing the target compounds [36]. MAE is very fast (often <30 min) and efficient, with the choice of solvent based on its dielectric properties being critical for success [35]. The combination of ultrasound and microwave techniques (UMAE) offers synergistic effects, often resulting in higher yields than either method alone [36]. Supercritical Fluid Extraction (SFE) uses a fluid, typically CO_2_, above its critical temperature and pressure and is uniquely suited for lipophilic phenolics in oil-rich matrices, offering high-purity, solvent-free extracts. In this state, the fluid has properties of both a liquid and a gas, allowing it to effuse through solids and dissolve materials [37]. While SFE is valued for its solvent-free operation and ability to prevent thermal degradation, its adoption is often limited by high equipment costs. In contrast, SCWE (or pressurized hot water extraction) offers a simpler, eco-friendly approach by using high-temperature water as a tunable solvent to extract a broad spectrum of analytes efficiently [38]. High Hydrostatic Pressure Processing (HHPP): This non-thermal method uses extremely high pressure (1000–8000 bar) to increase the permeability of cell membranes, enhancing the diffusion of cellular components into the solvent [39]. Like SFE or SPE (solid-phase extraction), it requires expensive specialized equipment.

## 4. Methods for Detection of Phenolic Compounds and Their Activity in Plants

**Figure 1:**
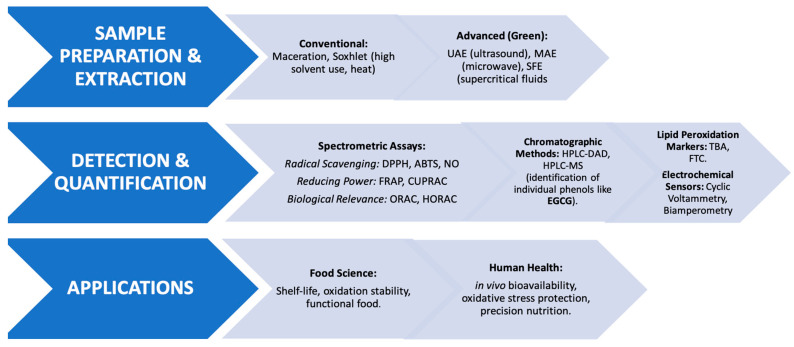
Integrated analytical workflow for the study of phenolic compounds and antioxidant activity.

Analytical techniques for quantifying phenolic compounds and antioxidant activity range from in vivo biological models to a diverse array of in vitro chemical assays. Key methodologies include radical quenching assays (DPPH—2,2-diphenyl-1-picrylhydrazyl, NO—Nitric Oxide Scavenging assay, and hydroxyl radicals), oxygen radical absorbance (ORAC—oxygen radical absorbance capacity, HORAC—hydroxyl radical averting capacity), and metal-reduction tests such as FRAP (test of the ferric reducing antioxidant power) and CUPRAC (cupric reducing antioxidant activity). Additionally, lipid peroxidation markers, like the TBA (Thiobarbituric Acid Reactive Substances) FTC (ferric thiocyanate) methods, are frequently employed to evaluate protective effects in complex matrices [40,41,42,43,44]. While the in vivo methods are as follows: ferric reducing ability of plasma, estimation of reduced glutathione (GSH) content, estimation of glutathione peroxidase (GSHPx), glutathione-S-transferase (GST), superoxide dismutase (SOD) method, Catalase (CAT), γ-glutamyl transpeptidase (GGT) activity assay, glutathione reductase (GR) analysis, lipid peroxidation (LPO) analysis, and LDL assay [45,46,47]. In the following chapters, the most commonly used methods for the determination of phenolic compounds and their antioxidant activity will be discussed. A description of the methodological framework is illustrated in Figure 1, which spans the innovative extraction technologies to a range of analytical tools and the subsequent effects on the quality of food and health of the population.

### 4.1. Spectrometric Techniques and Fluorometric Techniques

These assays are categorized based on their detection mode: Spectrophotometric (UV-Vis) assays (DPPH, ABTS, FRAP, CUPRAC) which measure color change, and Fluorometric assays (ORAC, HORAC) which measure fluorescence decay. Spectrometric techniques rely on the reaction of a radical, radical cation, or complex with an antioxidant molecule that is capable of donating a hydrogen atom. The DPPH method (2,2-diphenyl-1-picrylhydrazyl) uses a free radical that is stable due to the delocalization of the spare electron throughout the entire molecule [48]. The delocalization on the DPPH molecule determines the appearance of a purple color, with an absorption band with a maximum around 520 nm [49]. The reduction in DPPH by hydrogen donors results in a loss of its purple color [50], creating a linear relationship between absorbance decrease and antioxidant concentration. With Trolox serving as the benchmark standard [51], research indicates that while some compounds react rapidly based on their hydroxyl groups, the majority follow more complex, structure-dependent pathways [52,53,54]. Reaction speeds with DPPH vary greatly among antioxidants: ascorbic acid reacts very quickly, whereas BHT (Butylated Hydroxytoluene) requires an extended period of up to 90 min to reach completion. Propyl gallate shows a fast reaction rate, albeit slower than that of ascorbic acid [40]. Consequently, a 30 min standard was adopted for these measurements, highlighting that the timing of inhibition is a vital factor in determining antioxidant action [48]. Such protocols are designed to balance analytical sensitivity with external factors like pH, compound solubility, and the light-sensitive nature of DPPH [40,53]. The FRAP method is a test of the ferric reducing antioxidant power. The principle of this method is based on the reduction in the iron-tripyridyltriazine complex to its ferrous colored form in the presence of antioxidants [55]. The FRAP reagent contained 2.5 mL of a 10 mmol/L TPTZ (2,4,6-tripyridyl-s-triazine, Sigma) solution in 40 mmol/L HCl plus 2.5 mL of 20 mmol/L FeCl_3_ and 25 mL of 0.3 mol/L acetate buffer, pH 3.6, was freshly prepared and heated to 37 °C. Mix 40 μL of sample with 0.2 mL water and 1.8 mL FRAP reagent. After 10 min at 37 °C, measure absorbance at 593 nm using a 1 mmol/L FeSO_4_ standard [56,57]. The FRAP method depends on the reduction in the complex iron ion-TPTZ (2,4,6-tri(2-pyridyl) 1,3,5-triazine). A vibrant blue color forms when iron binds to the ligand; the darker the blue, the higher the antioxidant levels. This is measured against standards like Trolox or Vitamin C (ascorbic acid) for comparison [58,59].

The ORAC (oxygen radical absorbance capacity) analysis is a method that measures the scavenging activity of an antioxidant against the peroxyl radical, induced by 2,2′-azobis-(2-amidino-propane) dihydrochloride (AAPH), at 37 °C [49]. Fluorescein is used as a fluorescent probe. The loss of fluorescence is an indicator of the degree of degradation, a reaction with the peroxyl radical [54]. The HORAC (hydroxyl radical averting capacity) assay serves as an analytical tool to quantify the metal-chelating efficacy of antioxidants in environments that simulate Fenton-like chemistry [43]. The method relies on a Co(II) complex to monitor this inhibitory effect. The analytical protocol requires incubating the sample with fluorescein and then inducing radical generation via a Fenton mixture. Fluorescence levels are measured at one-minute intervals following an initial reading and agitation. Finally, results are calculated against a gallic acid standard curve [13,60,61].

A study was conducted on the effect of the extraction system on the extractability of polyphenolic compounds and the antioxidant action of various medicinal plants. The oxygen radical absorbance capacity (ORAC) and total polyphenol content of 25 Bulgarian medicinal plants subjected to extraction with water or an 80% acetone solution were tested and compared [62]. The type of extract significantly affected the efficiency of polyphenol extraction and antioxidant activity. In all cases, the ORAC results and total polyphenol content were higher for acetone extraction than for water extraction. The acetone extract of peppermint had the highest ORAC value—2917 μmol Trolox equivalent (TE)/g dry weight (DW) and polyphenol content—20,216 mg/100 g DW. For water extraction, thyme showed the highest ORAC antioxidant activity—1434 μmol TE/g DW [41,42,62]. A significant linear relationship was established between the concentration of total polyphenols and ORAC in the tested medicinal plants. It can be concluded that the solvent used significantly affects the polyphenol content and antioxidant activity of the extract, so for a better assessment of the antioxidant activity of natural products, it is recommended to use multiple extraction systems [54,62].

The TRAP (total radical-trapping antioxidant parameter) analysis uses chemiluminescence enhanced by luminols to monitor the reactions of the peroxyl radical. The CL signal is triggered by the production of luminol-labeled radicals, which is a result of the thermal decomposition of AAPH [52,60,61,63]. The TRAP value is determined based on the duration of the time period during which the sample quenched the chemiluminescence signal, due to the presence of antioxidants. The lipid peroxidation inhibition analysis is a procedure where a system similar to Fenton reactions (Co(II) + H_2_O_2_) is used to induce lipid peroxidation (e.g., fatty acids) [64]. The PFRAP (potassium ferricyanide reducing power) method is based on the increase in absorbance that can be related to the reducing ability of the antioxidant/antioxidant extracts. Compounds with antioxidant activity react with potassium ferricyanide, thereby forming potassium ferrocyanide. The latter reacts with ferric trichloride, yielding ferric ferrocyanide, a blue-colored complex, with a maximum absorption at 700 nm [65]. In the CUPRAC (cupric reducing antioxidant activity) method, standard antioxidants or extracts are mixed with CuSO4 and neocuproine. After 30 min, the absorbance is measured at 450 nm. In the assay, Cu(II) is reduced to Cu(I) by the action of electron-donating antioxidants. The results are expressed in milligrams of Trolox per liter of extract [44,66,67].

The fluorimetric analysis of phenolic compounds is grounded in the principle of fluorescence, where a substance emits electromagnetic radiation typically at a longer wavelength and lower energy level after absorbing incident light [68,69]. This phenomenon occurs when electrons, previously excited to a higher quantum state, release photons as they return to their ground state. Fluorescence spectroscopy has proven effective for profiling antioxidants within lipid matrices [69]. Specifically, this technique has been proposed for the quantification of butylated hydroxyanisole (BHA) and tert-butylhydroquinone (TBHQ) in biodiesel derived from sunflower and soybean oils. Experimental procedures involve recording excitation and emission spectra at room temperature [70,71]. typically, excitation at 310 nm produces an emission range of 320–800 nm. While native tocopherols in vegetable oils cause biodiesel to fluoresce near 420 nm, the introduction of BHA or TBHQ results in a distinct emission band at approximately 330 nm. Research confirms that the intensity of this 330 nm signal maintains a linear correlation with antioxidant concentration (R ≈ 1), a relationship that remains consistent regardless of the specific oil source [71]. The DPPH assay, for example, is affected by steric hindrance (large molecules cannot access the radical center), pH, and solvent polarity, causing the assay to underestimate the true antioxidant capacity. Conversely, the ORAC assay measures HAT (oriented around the center of the radical) and uses biologically relevant radicals, but is affected by endogenous fluorescence and temperature variations in complex matrices. The differences in the type of radicals and the underlying chemistry are the basis for the divergent results from the two assays.

### 4.2. Electrochemical Techniques

Electrochemical methods, notably cyclic voltammetry (CV), are effectively utilized for assessing antioxidant activity and concentration. This technique involves monitoring the current response while the working electrode potential undergoes a bidirectional linear scan, ultimately generating a cyclic voltammogram [72]. This voltammogram provides key parameters such as anodic and cathodic peak potentials (Ea, Ec) and currents (Ia, Ic) which reveal the antioxidant’s redox properties and concentration. CV is a validated method for quantifying the total antioxidant activity in complex samples like plant extracts, where the anodic current or wave area is used to measure the capacity, often expressed in equivalents of a standard like ascorbic acid [73]. The amperometric method involves measuring the current generated by the oxidation or reduction in an electroactive analyte at a fixed potential. In antioxidant analysis, this technique is often based on monitoring the reduction in a stable radical, such as DPPH (e.g., a glassy carbon electrode) [74]. Biamperometry relies on measuring the current between two identical working electrodes under a low potential difference. This technique effectively evaluates the antioxidant activity of both pure substances and complex matrices, such as wine and tea, frequently showing high correlation with traditional spectrophotometric assays [72]. For antioxidant analysis, it is typically used as an indirect measurement where the antioxidant analyte reacts with a reversible redox couple present in the solution. The change in the concentration of this redox couple is then detected. Commonly used indicating redox couples include Fe^3+^/Fe^2+^, I_2_/I^−^, and Fe(CN)_6_^3−^/Fe(CN)_6_^4−^ [73,74].

### 4.3. Biosensor Method

The field of analytical biotechnology has been significantly transformed by the development of biosensors, devices that integrate a biological recognition element with a physical transducer. Among the various biological components utilized, oxidoreductases have emerged as the gold standard, primarily due to their sophisticated mechanisms for electron transfer during catalytic cycles [75]. Potential applications of biosensors for assessing antioxidant status include monitoring the superoxide radical (O_2_^−^), nitric oxide (NO), glutathione, uric acid, ascorbic acid, or phenolic compounds [76]. A biosensor based on carbon paste was constructed for the electrocatalytic assessment of total antioxidant activity. The method was based on the partial damage to a DNA layer adsorbed on the electrode surface by OH radicals, generated by a Fenton reaction, and subsequent electrochemical oxidation of intact adenine bases, to create an oxidation product that could catalyze the oxidation of NADH [75,77]. The removal of hydroxyl radicals by antioxidants preserved adenine, increasing the NADH current signal (DPV). This method enabled the detection of ascorbic acid, used as a reference, at concentrations as low as 50 nM [76].

### 4.4. Chromatographic Methods

The content of phenolic compounds in plant material can be determined using several separation methods: high-performance liquid chromatography (HPLC), gas chromatography (GC), capillary zone electrophoresis (CZE), etc., as a set of individual compounds or by using specific chemical reactions as a group of chemically similar reactive compounds [78,79]. While chromatographic techniques offer high precision, the comprehensive identification of plant phenolics is often time- and cost-prohibitive; furthermore, HPLC-based antioxidant determination remains experimentally complex [80]. Phenolic content is highly variable, influenced by plant variety and external factors such as climate, cultivation practices, and post-harvest handling [81]. Consequently, spectrophotometric methods are widely used for the rapid assessment of total phenolics and antioxidant activity; phenolic profiling employs C18 columns with an acidified (0.1% formic acid) water + acetonitrile (or methanol) binary gradient. Detection uses DAD for class categorization and MS (ESI-QTOF or Triple Quad) for elucidation of DAD class structure. Analysts must deal with matrix effects, ion suppression, and co-elution of isomers [82,83]. However, standardized determination remains challenging due to the existence of over 20 different analytical methods. Discrepancies in experimental conditions and physicochemical properties make the direct comparison and general interpretation of results nearly impossible [84]. Ultimately, antioxidant efficacy depends heavily on the specific test system and the substrate involved. For volatile or vaporizable compounds, Gas Chromatography (GC) is employed, separating components between a gas mobile phase and a stationary phase for detection via retention times [85]. HPLC is a more versatile technique that uses a high-pressure pump to force a liquid mobile phase through a densely packed column containing a stationary phase. This allows for high-resolution separations. HPLC operates in two primary modes: normal-phase (polar stationary phase/non-polar mobile phase) and the more common reverse-phase (non-polar stationary phase/polar mobile phase) [86]. Compounds are identified by their characteristic retention times, and advanced detectors like a diode-array can provide additional spectral information. Antioxidant activity is determined using an HPLC system with an on-line post-column for antioxidant detection and is based on the radical scavenging action on 2,2′-azinobis-3-ethylbenzothiazoline-6-sulfonic acid [87].

HPLC-MS was utilized to identify isoflavone glycoside conjugates in soy products, with positive ion atmospheric pressure chemical ionization (APCI) providing optimal sensitivity and structural characterization [88]. While 80% methanol extraction is effective at room temperature, elevated temperatures (60–80 °C) should be avoided as they alter the isoflavone composition [89]. For tea analysis, HPLC-MS enabled the rapid profiling of over 30 phenols. Direct injection into reverse-phase HPLC using gradients optimized separately for catechins and flavonols/theaflavins allowed for efficient resolution [90,91,92]. Final identification relied on retention times, absorption spectra, and MS fragmentation patterns, encompassing a diverse range of catechins, theaflavins, glycosides, organic acids, and alkaloids [80].

Solid-phase extraction (SPE) was investigated by developing a method based on the prediction of retention data in HPLC or solvation parameters to determine the main parameters of any sequence (type and amount of sorbent, amount of sample that can be applied without loss, composition and volume of washing solutions, composition and volume of desorption solution) [93,94]. Obtaining extracts free of matrix noise is performed in several steps: the first step (when possible) represents the development of the SPE procedure. New selective phases are being reviewed, such as mixed modes with a restricted access module and sorbents or emerging phases, such as immunosorbents or molecularly imprinted polymers [95]. The selectivity of a combination of two sorbents with the use of a solution with an ion exchanger or ions was described. Special attention is paid to the complete automation of the SPE sequence with on-line coupling to liquid chromatography, and then to different detection modes. This represents a fast, modern, and reliable approach to the analysis of trace compounds [96]. The most important information and summary of extraction and analytical methods for phenolic compounds and antioxidant activity can be find in Table 1.

In a study, the BORWIN chromatographic system was used for HPLC data analysis. Chromatographic separation was performed using a Phenomenex C18 reverse-phase column (Phenomenex, 4.6 × 250 mm, 5 μm) at 25 °C, monitored at 340 nm. The linear gradient system with the solvent consisted of 75% solvent A and 25% solvent B, changed to 0% solvent A and 100% solvent B, with a flow rate of 0.5 mL/min, and the duration was 60 min [solvent A: 0.5% phosphoric acid in water; solvent B: 100% methanol]. To prepare stock solutions, extracts of ten flavonoids and kaempferol as an internal standard were dissolved in 100% MeOH at a concentration of 4 mg/mL and 2 mg/mL, respectively, and filtered through a centrifugal filter device (0.45 μm, Millipore Co., Bedford, MA, USA). The injection volume was 10 μL [81].

### 4.5. Comparisons of Different Methods

Comparative overview of antioxidant activity assays evaluated in this review can be find in the Table 2. However, in this chapter each of these methods will be explained in more detail. By analyzing a diverse set of 927 frozen vegetable specimens (ranging from 111 samples of white cabbage to 18 red peppers), researchers identified distinct discrepancies between ORAC and FRAP measurements. These variations are attributed to the underlying chemical mechanisms of each test. While ORAC is more chemically aligned with the behavior of chain-breaking antioxidants, FRAP is prone to issues like signal noise and inconsistent kinetics. The findings also suggest that environmental factors, such as origin and harvest season, play a crucial role in antioxidant activity, with spinach, beets, and cauliflower emerging as top performers under the ORAC protocol [100]. Investigating the antioxidant activity of tea, researchers found that ORAC and DPPH assays do not yield comparable results. Data indicate that while DPPH results align closely with catechin content (r = 0.99), ORAC results diverge (r = 0.7). A striking example is the performance of EGCG versus epicatechin across both platforms. The study attributes these variations to the structural properties of EGCG, specifically the pyrogallol moiety, which might act as a pro-oxidant under ORAC’s specific experimental conditions. This highlights a critical oversight in nutritional science: a low ORAC-to-DPPH ratio may signal pro-oxidant activity rather than simple antioxidant weakness, proving that these methodologies evaluate different chemical pathways [54].

The ORAC and TEAC analyses were compared to assess the total antioxidant activity (TAC) of orange juice, milk, and mixtures of orange juice and milk in the study of Zululeta et al., (2009) [41]. The sensitivity of antioxidant assays varies depending on the beverage components; for instance, while higher orange juice concentrations led to an increase in TAC across both methods, the TEAC assay failed to detect any increase when milk content was raised. In contrast, the ORAC method successfully captured the additional antioxidant contribution from both juice and milk. This suggests that TEAC, although more economical and straightforward, may provide a less accurate representation of antioxidant activity in chemically diverse samples. The study further evaluated how individual constituents, including albumin, ascorbic acid, and various carotenoids like lutein and β-carotene, influence these overall measurements [41].

The antioxidant activities measured in a methanol extract obtained using ABTS, DPPH, FRAP, and ORAC analyses of a single extract were measured three times to determine the reproducibility of the tests [81,99,101]. The DPPH and FRAP tests showed no differences between determinations, while the ABTS and ORAC tests differed in different cycles. All tests, however, had no genotype-by-time interaction, indicating that all techniques gave a comparable rank of antioxidant action among replications within each determination time [102]. The preparation time for antioxidant assays varies significantly; DPPH, FRAP, and ORAC can be applied immediately, whereas the ABTS method requires a 12 h dark-storage period to activate the radical cation from its salt precursor. Once activated, the ABTS reagent’s stability is restricted to 4 h. Despite these procedural requirements, ABTS, along with the highly reproducible DPPH and FRAP tests, remains a key tool for determining the antioxidant activity of guava fruits [103]. Since the working sample of ABTS is not always the same age, the activity of the reaction mixture with guava extracts could be different at the time of determination. The reading value was higher at the top than at the bottom and also from the left side to the right side [104]. A smaller coefficient of variance (CV) is obtained using a 48-well plate compared to the 96-well format. The 48-well plate format had a CV of about 50% of the CV of the data generated in the 96-well plate. Thus, an increased number of sample wells on the plate induced an increased error rate in the tests [105,106]. In terms of the cost and time of performing these methods, the main disadvantage of the ORAC technique is that it requires the use of expensive equipment, while the other three methods require simpler apparatus and a spectrophotometer that is common in most laboratories [107]. Another advantage of the ABTS and FRAP methods is that the extracts reacted quickly with ABTS (2 h) or the iron ion (30 min), while the DPPH reaction took much longer (24 h) [42].

## 5. Discussion

The analytical landscape of phenolic compounds is currently undergoing a radical transformation, moving beyond the mere quantification of total content toward a multifaceted understanding of their biological efficacy and environmental footprint [108]. While traditional colorimetric assays such as DPPH and FRAP remain ubiquitous due to their operational simplicity, the current consensus in the scientific community (2023–2025) suggests they are increasingly insufficient as standalone metrics for high-impact research. These methods are fundamentally limited by their inability to reflect the complex redox signaling and metabolic transformations that occur in vivo. Consequently, a paradigm shift is occurring where “antioxidant activity” is being replaced by the concept of “Redox Fingerprinting”, which integrates chemical profiling with bioaccessibility data [109]. This evolution is driven by the urgent need for standardization, as the current lack of a universal analytical protocol continues to hamper the comparability of results across global studies [109,110]. As a point of fact, the DPPH, FRAP, and ORAC methods each measure certain aspects of redox potential, and do not actually measure the biological activity or effectiveness of a compound. They measure a compound’s “Total Antioxidant Potential” instead of health and biological activities. FRAP, while favorable in rapid quality control, omits thiol activity. On the other hand, ORAC is considered more relevant biologically, but still lacks the layer of complexity that is present in cells. Consequently, these assays are better used as part of a larger whole, with the aim of integrating both the single electron transfer (SET) and the hydrogen atom transfer (HAT) to obtain a more complete view of the antioxidant activity in question. A critical frontier in this field is the transition to Green Extraction 4.0, where toxic organic solvents are being phased out in favor of Natural Deep Eutectic Solvents (NADES) and subcritical water technologies. Recent studies (2024–2025) demonstrate that NADES do not merely act as extractants but serve as “protective scaffolds” that stabilize sensitive phenolic structures through hydrogen bonding, thereby enhancing their shelf-life and biological potency [111,112]. This synergy between extraction and stability is vital for the development of next-generation functional foods. As for comparability, explicit parameters for reporting extraction need to include the solvent ratios, temperature, and time. Results must be normalized to dry weight (DW) and rather than expressed as reduction percentages, must be presented in stable standards (e.g., Trolox or gallic acid equivalents). Lastly, all runs must include solvent blanks, positive controls, and at least three technical and two biological replicates. Furthermore, the integration of Artificial Intelligence (AI) and Machine Learning (ML) is now enabling researchers to bypass the labor-intensive “trial and error” approach. New AI models [113] can finally predict how complex mixtures of antioxidants interact, bypassing the errors common in older lab methods [110,111]. Looking toward the 2026–2030 horizon, the focus must shift from the food matrix to the intestinal ecosystem. The true antioxidant potential of phenolics is not realized in the test tube, but rather after microbial transformation in the gut. Modern research is increasingly adopting the INFOGEST 2.0 protocol and organ-on-a-chip technologies to track the conversion of complex polyphenols into highly bioactive metabolites, such as urolithins and phenolic acids [114,115,116]. This biological bridging is essential to overcome the “antioxidant paradox” where high in vitro activity fails to manifest in clinical benefits. Concurrently, the rise of portable sensing technology, specifically electrochemical paper-based devices (ePADs) integrated with smartphone AI, is democratizing phenolic analysis, allowing for real-time monitoring of food quality and antioxidant status at the farm or production line [117,118]. Ultimately, the future of phenolic research lies in precision nutrition instead of generic dietary recommendations; the field is moving toward tailoring phenolic intake to an individual’s specific redox status and microbiome composition. In response to the urgent need for standardized methodology, and to offer a constructive approach for forthcoming research, we propose the implementation of a minimum reporting and quality control (QC) protocol that specifies the following sample pretreatment (e.g., drying and defatting), extraction (solvent ratio, time, temperature), and calibration standards adjusted to dry weight with regard to Trolox or Gallic Acid Equivalents, as well as a comprehensive approach with respect to experimental design, inclusive of solvent blanks, positive control(s), and a clearly defined replicated control.

## 6. Conclusions and Future Perspective

The analysis of phenolic compounds and their antioxidant activity is fundamentally limited by a lack of methodological standardization. Variability in sample preparation and extraction, coupled with the use of numerous in vitro assays (e.g., DPPH, FRAP, ORAC) based on disparate chemical principles, makes direct comparison of results between studies problematic and obscures their true biological relevance. Future progress in the field requires a clear paradigm shift. This involves adopting greener and more efficient extraction techniques like UAE, MAE, and SFE, while moving beyond simple total activity measurements towards comprehensive profiling with advanced analytical tools like HPLC-MS. The ultimate solution lies in an integrated approach that combines a battery of chemical assays with more biologically relevant models, such as cell-based tests and metabolomics. This strategy will bridge the gap between in vitro measurements and in vivo effects, providing a more accurate understanding of the role of phenolic compounds in food and health. To improve practicality, a few suggestions and recommendations may be made. For instance, anthocyanins, which are sensitive compounds, prefer UAE (<40 °C), while MAE is better for stable phenolic acids in hard tissues. In assays, longer incubation times are suggested for FRAP (30 min) and for DPPH (up to 24 h) to capture slowly reacting polyphenols. For a thorough redox profile, an integrated approach with SET-based (FRAP) and HAT-based (ORAC) assays is critical. Lastly, in order to improve reproducibility and comparisons to be made to other studies, results should be standardized to Trolox or Gallic acid equivalents. By combining high-resolution chromatography, green extraction, and AI-driven predictive modeling, we can finally bridge the gap between chemical quantification and human health outcomes. The future researcher must therefore view phenolic analysis not as a static measurement, but as a dynamic dialogue between the environment, the food matrix, and the human host.

## Figures and Tables

**Table 1:** Summary of extraction and analytical methods for phenolic compounds and antioxidant activity in the reviewed literature.

Category	Method/Technique	Principle/Description	Key Remarks	References
Extraction	Conventional (Soxhlet, Maceration)	Uses solvents over extended periods, often with heat. Simple, inexpensive apparatus. (seeds, nuts, cereals)	Long extraction times, risk of thermal degradation of compounds, high solvent consumption.	[22,24,62]
	Ultrasound-Assisted (UAE)	Uses ultrasonic waves (>20 kHz) to disrupt plant cell walls and enhance solvent penetration. (leaves, soft fruits)	Simple, inexpensive, and rapid. Improves mass transfer.	[36]
	Microwave-Assisted (MAE)	Uses microwave energy to heat the solvent and intracellular water, causing cell rupture. (seeds, bark, roots)	Very fast (<30 min) and efficient. Solvent choice is critical (dielectric properties).	[97,98]
	Supercritical Fluid (SFE)	Uses a fluid (typically CO_2_) above its critical point, giving it gas and liquid properties. (grape seed, oils)	Highly selective, avoids toxic organic solvents, prevents thermal degradation. High equipment cost.	[37,40,48]
Spectrometric	DPPH	Measures the scavenging of the stable 2,2-diphenyl-1-picrylhydrazyl radical, observed by a color change from purple to yellow. (fruits, beverages)	Simple and common, but reaction kinetics can be slow and vary between antioxidants.	[40,48]
	FRAP	Measures the ability of an antioxidant to reduce a ferric-tripyridyltriazine (Fe^3+^-TPTZ) complex to its ferrous (Fe^2+^) form.	Quick and simple, but does not measure the activity of all types of antioxidants (e.g., thiols).	[55,58]
	ORAC	Measures the inhibition of peroxyl radical-induced oxidation of a fluorescent probe (fluorescein). (complex foods like milk and juice)	Considered more biologically relevant as it uses a biologically relevant radical source. Requires expensive equipment.	[41,99]
	HORAC	Measures the metal-chelating ability of an antioxidant, assessing protection against hydroxyl radical formation. (beverages)	Based on a Fenton-like reaction; uses gallic acid as a standard.	[43]
	CUPRAC	Measures the reduction in cupric ions (Cu^2+^) to cuprous ions (Cu^+^) by antioxidants in the presence of neocuproine.	Applicable to a wide range of antioxidants, including hydrophilic and lipophilic ones.	[44,67]
Electrochemical	Cyclic Voltammetry (CV)	Scans the potential of an electrode and records the resulting current to determine the redox properties of antioxidants.	Provides information on the antioxidant’s redox potential and concentration.	[74]
Chromatographic	HPLC/HPLC-MS	Separates individual compounds based on their interaction with a stationary phase, followed by detection (e.g., UV, mass spectrometry).	Precise and accurate for identifying and quantifying individual phenolic compounds, but complex and costly.	[80,81]

**Table 2:** Comparative overview of antioxidant activity assays evaluated in this review.

Assay	Key Advantage(s)	Key Disadvantage(s)	Comparative Insights & Remarks	References
ORAC	High Biological Relevance: Considered more chemically relevant for chain-breaking antioxidants. Effective in Complex Matrices: Accurately reflects antioxidant activity in mixtures like juice and milk.	High Cost: Requires expensive, specialized equipment (fluorometer). Lower Reproducibility: Showed greater variability between cycles compared to DPPH and FRAP. Pro-oxidant Interference: Can yield misleadingly low results for compounds that exhibit pro-oxidant behavior (e.g., EGCG).	Exhibits a weak correlation with total polyphenol content in tea (r ≈ 0.7) compared to DPPH. Its discrepancy with other methods can be an indicator of a compound’s potential pro-oxidant activity.	[51,54,99]
FRAP	High Reproducibility: Showed no significant differences between repeated determinations. Very Rapid: Features a fast reaction time of approximately 30 min. Simplicity & Low Cost: Uses standard laboratory spectrophotometers.	Lower Chemical Relevance: Prone to issues with signal noise, reaction kinetics, and quantification compared to ORAC.	Due to its high reproducibility and speed, it is a reliable method for routine analysis, often paired with DPPH.	[42,58,59]
DPPH	High Reproducibility: Consistently reliable across repeated measurements. Strong Correlation with Phenolics: Showed an excellent correlation with total polyphenol and catechin content in tea (r ≈ 0.99).	Extremely Slow Reaction Time: The reaction requires up to 24 h for completion, limiting its practicality for high-throughput screening.	Although highly reproducible, the long incubation period is a significant practical drawback.	[40,42,51]
TEAC (ABTS)	Simpler & Lower Cost than ORAC. Faster than DPPH: Reaction time is approximately 2 h.	Lower Reproducibility: Exhibited variability in results between cycles. Long Reagent Preparation: Requires a 12 h incubation to generate the radical, which introduces a source of variability. Underestimates in Complex Samples: May report lower antioxidant activity in chemically complex food and beverage matrices.	The accuracy is highly dependent on the age and stability of the working reagent.	[41,99]

## Data Availability

The data presented in this study are available within the article (Review). No new raw data were created or analyzed in this study.
